# Role of TSPO/VDAC1 Upregulation and Matrix Metalloproteinase-2 Localization in the Dysfunctional Myocardium of Hyperglycaemic Rats

**DOI:** 10.3390/ijms21207432

**Published:** 2020-10-09

**Authors:** Micaela Gliozzi, Federica Scarano, Vincenzo Musolino, Cristina Carresi, Miriam Scicchitano, Stefano Ruga, Maria Caterina Zito, Saverio Nucera, Francesca Bosco, Jessica Maiuolo, Roberta Macrì, Lorenza Guarnieri, Rocco Mollace, Anna Rita Coppoletta, Caterina Nicita, Annamaria Tavernese, Ernesto Palma, Carolina Muscoli, Vincenzo Mollace

**Affiliations:** 1 IRC-FSH Department of Health Sciences, University “Magna Græcia” of Catanzaro, 88100 Catanzaro, Italy; federicascar87@gmail.com (F.S.); v.musolino@unicz.it (V.M.); carresi@unicz.it (C.C.); miriam.scicchitano@hotmail.it (M.S.); rugast1@gmail.com (S.R.); mariacaterina.zito@libero.it (M.C.Z.); saverio.nucera@hotmail.it (S.N.); boscofrancesca.bf@libero.it (F.B.); jessicamaiuolo@virgilio.it (J.M.); robertamacri85@gmail.com (R.M.); lorenzacz808@gmail.com (L.G.); rocco.mollace@gmail.com (R.M.); annarita.coppoletta@libero.it (A.R.C.); caterinanicita@libero.it (C.N.); palma@unicz.it (E.P.); muscoli@unicz.it (C.M.); mollace@libero.it (V.M.); 2 Renato Dulbecco Institute, Presso Fondazione Terina, 88046 Lamezia Terme (CZ), Italy; an.tavernese@gmail.com; 3 Division of Cardiology, University Hospital Policlinico Tor Vergata, 00133 Rome, Italy; 4 IRCCS San Raffaele Pisana, Via di Valcannuta, 00163 Rome, Italy

**Keywords:** hyperglycaemia, superoxide anion, mitochondrial dysfunction, TSPO, VDAC1, MMP-2, MAM, cardiomyocyte, diabetes, myocardium

## Abstract

Clinical management of diabetic cardiomyopathy represents an unmet need owing to insufficient knowledge about the molecular mechanisms underlying the dysfunctional heart. The aim of this work is to better clarify the role of matrix metalloproteinase 2 (MMP-2) isoforms and of translocator protein (TSPO)/voltage-dependent anion-selective channel 1 (VDAC1) modulation in the development of hyperglycaemia-induced myocardial injury. Hyperglycaemia was induced in Sprague-Dawley rats through a streptozocin injection (35 mg/Kg, i.p.). After 60 days, cardiac function was analysed by echocardiography. Nicotinamide Adenine Dinucleotide Phosphate NADPH oxidase and TSPO expression was assessed by immunohistochemistry. MMP-2 activity was detected by zymography. Superoxide anion production was estimated by MitoSOX™ staining. Voltage-dependent anion-selective channel 1 (VDAC-1), B-cell lymphoma 2 (Bcl-2), and cytochrome C expression was assessed by Western blot. Hyperglycaemic rats displayed cardiac dysfunction; this response was characterized by an overexpression of NADPH oxidase, accompanied by an increase of superoxide anion production. Under hyperglycaemia, increased expression of TSPO and VDAC1 was detected. MMP-2 downregulated activity occurred under hyperglycemia and this profile of activation was accompanied by the translocation of intracellular N-terminal truncated isoform of MMP-2 (NT-MMP-2) from mitochondria-associated membrane (MAM) into mitochondria. In the onset of diabetic cardiomyopathy, mitochondrial impairment in cardiomyocytes is characterized by the dysregulation of the different MMP-2 isoforms. This can imply the generation of a “frail” myocardial tissue unable to adapt itself to stress.

## 1. Introduction

Diabetic cardiomyopathy (DCM) represents a complication of diabetes and is a relevant cause of hospitalization and mortality among affected patients. However, despite the incidence of DCM, to date, its pathophysiological mechanisms still remain unclear.

Clinical data show that ventricular hypertrophy as well as diastolic and systolic dysfunction are the main detrimental events occurring as a result of chronic hyperglycaemia, even in those patients with well-controlled blood glycaemic levels [1,2,3]. Although DCM is commonly considered the direct consequence of coronary artery disease and hypertension, it is well defined by now that it can develop independently from macro- and micro-vascular diseases [4].

In particular, growing evidence identifies the coexistence of mitochondrial dysfunction and pathological extracellular matrix remodelling as the leading cause of myocardial injury [5].

The emerging role of dysfunctional mitochondria in the onset and development of DCM suggest that the intracellular levels of reactive oxygen species (ROS) are tightly correlated with the entity of myocardial damage. Accordingly, recent discoveries highlighted the importance of the superoxide anion (O_2_^−^) amount [6,7] in the determination of cardiomyocyte fate between survival or apoptosis. In hyperglycaemia-induced cardiovascular complications, NADPH oxidase represents the main source of mitochondrial O_2_^−^, which is a fundamental regulator in the formation of transition pore. Indeed, the O_2_^−^ amount can mediate the association between 18 kDa translocator protein (TSPO) and voltage-dependent anion-selective channel 1 (VDAC1), in order to prevent mitochondrial dysfunction or to promote apoptosis [8].

The excessive deposition of extracellular matrix (ECM) is a contributory cause responsible for the impairment of altered myocardial tissue geometry and function. Indeed, all the alterations regarding the architecture of a diabetic heart derive from those molecular mechanisms converging in the pathogenesis of heart fibrosis [5,6,7,9].

Matrix metalloproteinases (MMPs) are recognized as the main enzymes involved in extracellular matrix degradation and remodelling in both physiological and pathological states, although their role in the progression of DCM remains not entirely clarified [10]. Among them, MMP-2 plays a very important role in the process of physiological or pathological cardiac remodelling and appears to be involved, at different levels, in the development of cardiac disease [11]. Particularly, its action is tightly related to the biochemical status of the protein, which can appear in three different isoforms: (1) secreted MMP-2, able to regulate the extracellular matrix compartments; (2) intracellular MMP-2, which is a latent, full length form associated with the sarcomeric contractile apparatus; and (3) intracellular N-terminal truncated isoform of MMP-2 (NT-MMP-2), produced under hypoxia and oxidative conditions [11,12].

Despite the progress of knowledge regarding MMPs’ function and the discovery of additional factors contributing to DCM development, such as impaired calcium homeostasis [13], ultrastructural changes [14], and advanced glycation end products (AGE) [15], poor evidence exists correlating mitochondrial dysfunction, ROS production, and these additional features [16]. Moreover, although the role of chronic hyperglycaemia is considered the primary pathophysiological stimulus responsible for myocardial injury, the effects of impaired glucose levels in the early stages of DCM remain to be elucidated. In this consideration, our study highlights the role of MMP-2 in the regulation of mitochondrial injury mediated by TSPO and VDAC1 in the onset of DCM, thus contributing to the identification of novel potential targets of cardioprotective therapeutic interventions under hyperglycaemia.

## 2. Results

### 2.1. Glycaemia Evaluation

A significantly increase of blood glucose levels was observed in animals compared with the normal pellet diet (NPD) group after two weeks from streptozocin injection (*p* < 0.001). The significant increase of hematic glucose persisted until the end of the experiment (Supplementary Figure S1).

### 2.2. Hyperglycaemia Induces an Impairment of Myocardial Contractility

**Figure 1:**
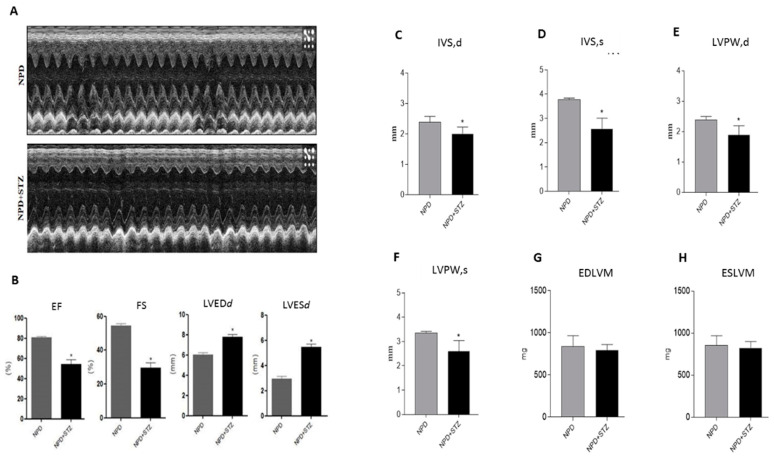
Effects of hyperglycaemia on left ventricle (LV)-dysfunction. (**A**) Representative monodimensional echocardiographic imaging of normal pellet diet (NPD) and NPD + streptozotocin (STZ) groups at the end of the study. (**B**) Histograms show cardiac dimensions and functional parameters assessed at the end of the study. Hyperglycemia in NPD + STZ rats significantly increased LV end-systolic diameter (LVES*d*) and LV end-diastolic diameter (LVED*d*), impairing fractional shortening (FS) and ejection fraction (EF) as compared with the NPD group. (**C**,**D**) In NPD + STZ animals, a significant thinning of interventricular septum in systole (IVSs) and in dyastole (IVS*d*) and (**E**,**F**) the reduction of left ventricular posterior wall in systole (LVPW*s*) and in diastole (LVPW*d*) were detected in comparison with the NPD group. (**G**,**H**) No differences in end diastolic left ventricular mass (EDLVM) and in end systolic left ventricular mass (ESLVM) were observed between the two groups. The data are presented as mean ± SEM. * *p* < 0.05 vs. NPD, Mann–Whitney test, *n* = 6/group.

Echocardiographic analysis shows that hyperglycemia (normal pellet diet (NPD) + streptozotocin (STZ)) significantly increased the diameter of the left ventricular chamber in diastole (LVEDd) and in systole (LVESd) as compared with NPD hearts (Figure 1A). These pathological conditions were associated with a reduced ejection fraction (EF) and fractional shortening (FS) in relation to the NPD group (Figure 1B).

The analysis of the left ventricular wall thicknesses revealed a thinning of the interventricular septum (IVS) and the left ventricular posterior wall (LVPW), in both diastole and systole, in NPD + STZ hearts compared with the NPD group (Figure 1C–F). No alterations in end diastolic left ventricular mass (EDLVM) and in end systolic left ventricular mass (ESLVM) were observed between the two groups (Figure 1G–H).

**Figure 2:**
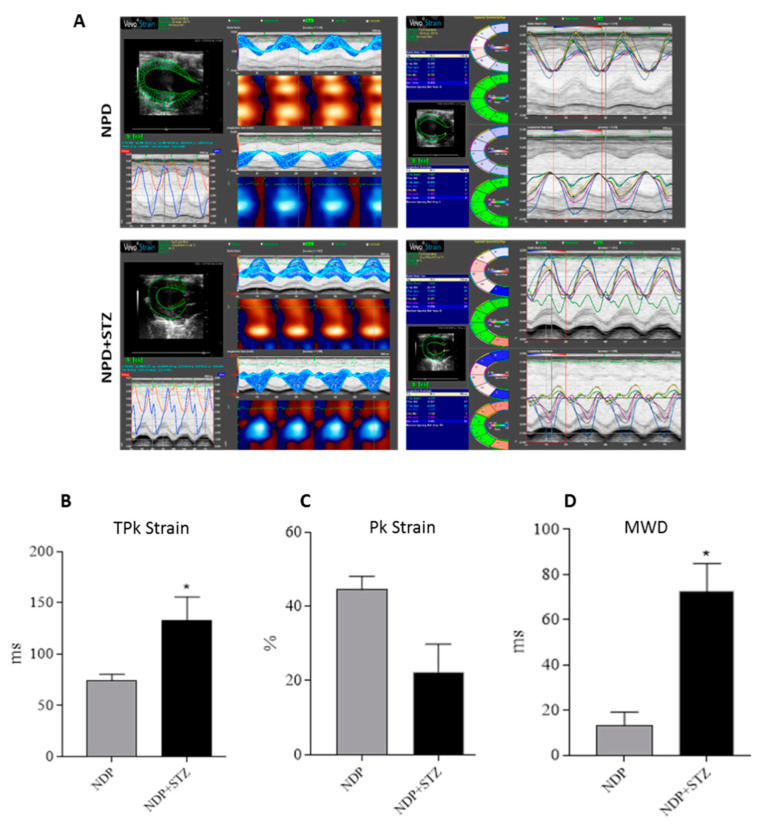
Cardiac tissue strain analysis. (**A**) Representative parametric displays, area/volume graphs, and segmental synchronicity page related to endocardial strain analysis of parasternal short-axis view (PSLAX) assessed in radial motion at the end of experimental period. (**B**) Average of time-to-peak (TPk) of the six segments of cardiac wall in endocardial strain analysis. (**C**) Whole peak capacity, assessed as the peak average (Pk) of the six segments of cardiac wall, in endocardial strain. (**D**) Maximum wall delay (MWD) of LV during radial motion in endocardial strain analysis. The data are presented as mean ± SEM. * *p* < 0.05 vs. NPD, Mann–Whitney test, *n =* 6/group.

The subsequent global analysis of strain of the LV at the endocardium in the radial motion indicated that the average of time to reach the peak (TPk) of the six segments of cardiac wall was increased in NPD + STZ treated rats compared with that of the NPD group (Figure 2B). On the other hand, NPD + STZ treated rats whole peak capacity, assessed as the peak average (Pk) of the six segments of cardiac wall, showed a decrease in endocardial strain analysis (Figure 2C) when compared with the NPD group.

For the regions analysed, the hearts of NPD + STZ treated rats showed a larger time delay between the fastest and the lowest region (maximum wall delay—MWD), which leads to an overt dyssynchrony in cardiac wall motion when compared with the NPD group. Indeed, through the strain analysis, a significantly increased endocardial MWD (Figure 2D) was assessed when compared with the NPD group.

### 2.3. Hyperglycaemia Causes NADPH Oxidase-Induced Superoxide Anion Formation, as well as TSPO and VDAC1 Overexpression

**Figure 3:**
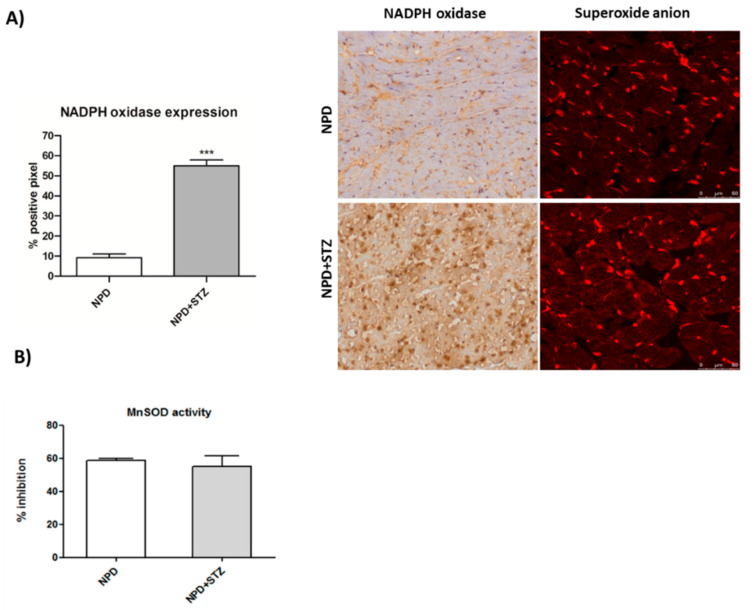
Effect of hyperglycaemia on NADPH oxidase expression and superoxide dismutase (SOD) activity in myocardial tissue. (**A**) In the STZ group, up-regulation of NADPH oxidase was associated with higher detection of superoxide anion, compared with the NPD group. On the left, quantification of NADPH expression related to immunohistochemistry analysis and representative confocal images showing MitoSOX™ staining. Red fluorescence indicates the formation of superoxide anion. (**B**) SOD activity in mitochondrial fraction remained unchanged between the two groups. The data are presented as mean ± SEM. *** *p* < 0.001 vs. NPD, Mann–Whitney test, *n* = 6/group.

NADPH oxidase expression significantly increased in myocardial tissue under hyperglycaemia (Figure 3A). Moreover, it was associated with enhanced levels of superoxide anion as compared with the NPD group (Figure 3A), although no changes in mitochondrial superoxide dismutase (SOD) activity were revealed (Figure 3B).

**Figure 4:**
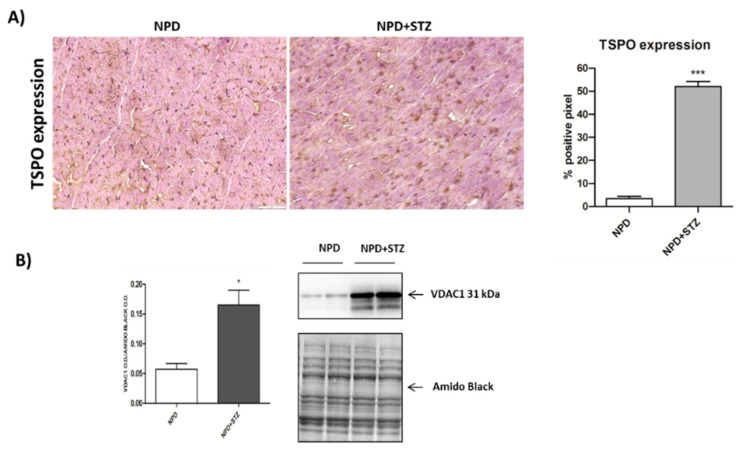
Effect of hyperglycaemia on translocator protein (TSPO) and voltage-dependent anion-selective channel 1 (VDAC1) expression in myocardial tissue. (**A**) On the left, representative images showing that, in the STZ group, an up-regulation of TSPO expression was observed in comparison with the NPD group. On the right, quantification of TSPO expression related to immunohistochemistry analysis. (**B**) Densitometric analysis of VDAC1 expression assessed by Western blot assay showing the enhancement of protein level in mitochondrial fraction under hyperglycemia (on the right, representative images of Western blot) The data are presented as mean ± SEM. * p < 0.05; *** *p* < 0.001 vs. NPD; Mann–Whitney test, *n* = 6/group (immunohistochemistry analysis); *t*-test, *n* = 6/group (Western blot analysis).

Finally, TSPO expression was up-regulated in the hearts of NPD + STZ rats compared with NPD cardiac tissue (Figure 4A).

**Figure 5:**
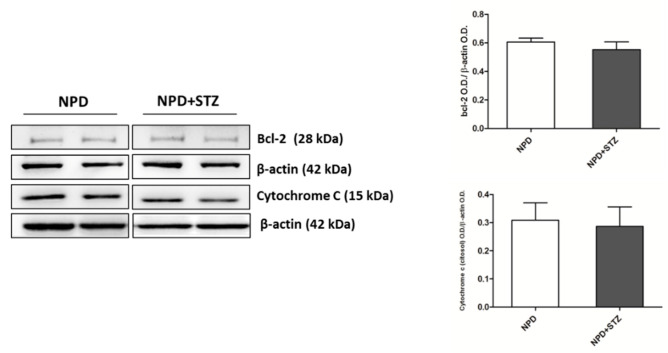
Bcl-2 and cytochrome C expression in hyperglycaemic and normoglycaemic animals. Western blot analysis showed no changes in Bcl-2 and cytochrome C expression between the two groups. On the left, representative images of Western blot assay; on the right, densitometric analysis. The data are presented as mean ± SEM. *n* = 6/group *(t*-test).

The enhancement in TSPO levels was associated with the overexpression of VDAC1 in mitochondrial fraction (Figure 4B). Differently, cytosolic Bcl-2 and cytochrome C expression did not change between the NPD + STZ and NPD groups (Figure 5).

### 2.4. Effects of Hyperglycaemia on the Activity and Localization of the Different MMP-2 Isoforms

**Figure 6:**
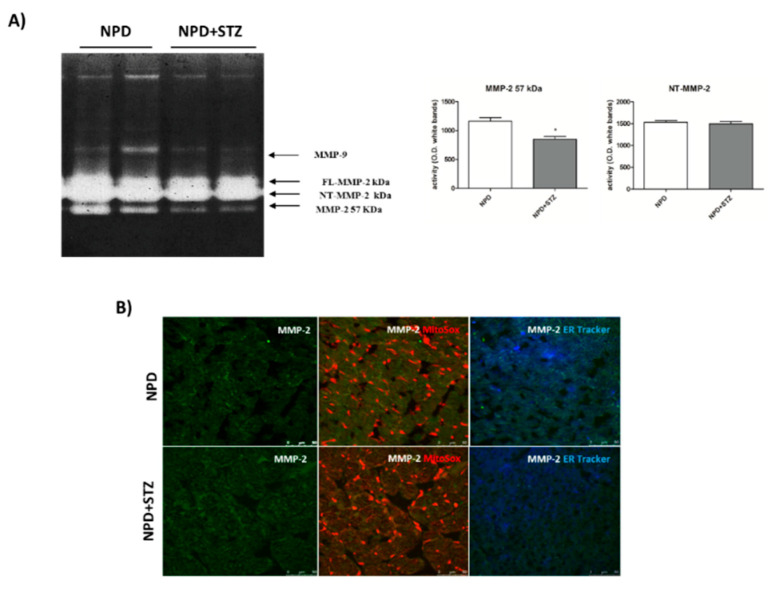
Effect of hyperglycaemia on MMP-2 activity and localization in myocardial tissue. (**A**) Zymogram showed a decreased activation of the extracellular MMP-2 (57 kDa) in NPD + STZ rats compared with the NPD group, whereas no difference of full-length FL-MMP-2 and N-terminal truncated isoform of MMP-2 (NT-MMP-2) activities was detected. (**B**) Representative images of immunofluorescence analysis. Orange staining is due to colocalization of mitochondria (MitoSOX™-induced coloration) with NT-MMP-2 (green). Blue/white staining is due to colocalization of endoplasmic reticulum (ER-Tracker™-induced blue) with NT-MMP-2 (green). * *p* < 0.05; vs. NPD. The data are presented as mean ± SEM, *t*-test *n* = 6/group.

NPD + STZ animals showed a decreased activation of myocardial extracellular MMP-2 (57 kDa) as compared with the control group. No differences in FL-MMP-2 and NT-MMP-2 activities between the two groups were detected (Figure 6A). Despite the unchanged activity, immunofluorescence analysis showed a mitochondrial accumulation of NT-MMP-2 in NPD + STZ rats in comparison with the NPD group. This result was associated with a higher ER-localization in the myocardium of NPD animals (Figure 6).

### 2.5. The Gelatinolytic Activity of MMP-2 is Prevented by Ca^2+^ Chelation

In situ zymography assessed on heart sections shows that gelatinolytic activity mainly attributable to MMP-2 activation was down-regulated in NPD + STZ rats as compared with the NPD group. The activation was prevented in the presence of the chelating agent Ethylenediaminetetraacetic acid EDTA (Supplementary Figure S2).

## 3. Discussion

**Figure 7:**
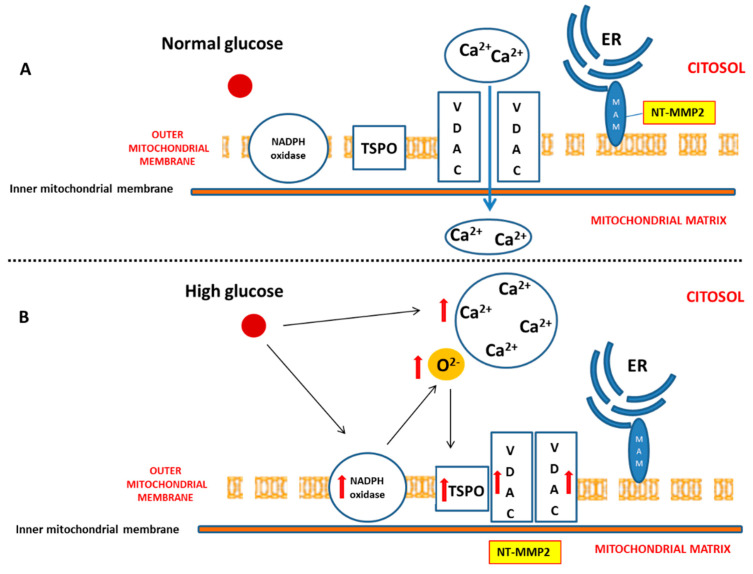
Effect of hyperglycaemia on cardiomyocyte. (**A**) Under physiologic glucose levels, VDAC1 promotes mitochondrial calcium intake and buffering. (**B**) High glucose levels directly induce an increase of cytosolic calcium. Moreover, chronic hyperglycaemia causes mitochondrial NADPH overexpression and superoxide anion overproduction, thus promoting TSPO and VDAC1 overexpression. This, in turn, results in their probable interaction aimed to prevent mitochondrial calcium accumulation. On the other hand, enhanced cytosolic calcium promotes endoplasmic reticulum (ER) impairment, translocation of NT-MMP-2 from mitochondria-associated membrane (MAM) into mitochondrial matrix, and mitochondrial dysfunction.

Diabetes mellitus (DM) is considered a risk factor for heart failure, and the enhancement of reactive oxygen species (ROS) production represents the major cause of cardiac dysfunction in patients with DM [6,17]. Our results confirm the importance of the NADPH oxidase-dependent production of superoxide anion in a diabetic heart, which is able to gradually compromise myocardial structure and function. In particular, we have demonstrated that, during the early phase of cardiac injury caused by chronic hyperglycaemia, ventricular dysfunction was associated with a change in the outer mitochondrial membrane, characterized by TSPO and VDAC1 overexpression. Moreover, we hypothesize that the interaction between TSPO and VDAC1, probably impeding mitochondrial calcium uptake, contributes to determining endoplasmic reticulum (ER) stress and the translocation of the intracellular NT-MMP-2 into mitochondria, thus causing an impairment of the function of these organelles. In this context, we have also demonstrated for the first time that the driving cause of ventricular dysfunction in diabetic heart depends on NT-MMP-2 intracellular localization, affecting mitochondria, rather than on the enhanced degradation of ECM (Figure 7).

The clinical management of diabetes-induced cardiovascular disease remains an unresolved problem because of the insufficient knowledge regarding the molecular mechanisms underlying the process of cardiac dysfunction [18]. As a consequence, to date, cardiovascular disorders still represent the primary cause of morbidity and mortality among all the complications deriving from diabetes [6].

In this scenario, several pathologic mechanisms leading to cardiac dysfunction have been hypothesized, and most of them have been associated with mitochondrial injury, which is considered a very important etiopathological cause of diabetic heart disease [5,19,20].

Commonly, mitochondrial dysfunction is characterized by an overproduction of reactive oxygen species that, triggering the release of pro-apoptotic factors, such as cytochrome C, determines cardiomyocyte death, thus impairing the function of myocardium [21,22]. However, although the use of antioxidant molecules has demonstrated an amelioration of cardiac damage in experimental models of diabetes [22,23], it has not shown any relevant protective effect in diabetic patients [24,25], suggesting the existence of additional mechanisms underlying DCM [5]. This discrepancy might be due to a variable amount of free radicals depending on the coexistence of other comorbidities [5,26]. Indeed, our previous and not fully published data show that a chronic injury predisposing to higher free radical production, such as a high fat diet, accelerates mitochondrial membrane oxidation, determining an initial cytochrome c release that, in the early stage of diabetic cardiomyopathy, is counteracted by enhanced expression of bcl-2 [26]. These events are not accompanied by the formation of cleaved caspase-3 (unpublished data), in accordance with other discoveries highlighting that the involvement of cytochrome c in apoptosis begins much earlier than the formation of apoptosome and caspase activation. In particular, this process starts with cardiolipin oxidation, catalysed by a cardiolipin-specific cytochrome c activated by low levels of ROS [27], and can be characterized by Bcl-2 and Bax overexpression, no signs of caspase activation, and by cell survival with significant amounts of cytochrome c in the cytoplasm [28]. Our results, supported by other previous discoveries [26,29], also show that hyperglycaemia associated with cardiac dysfunction is able to induce an enhancement of NADPH oxidase expression, which is not associated with any alteration of SOD activity. This confirms that constant and slight increased levels of O_2_^−^ represent an initial important cause of myocardial dysfunction in a diabetic heart. Thus, in this context, the amount of ROS production has been reconsidered as a relevant mediator of apoptotic programme rather than the main result of mitochondrial dysfunction and impairment [27].

Additional evidence shows that NADPH oxidase-induced ROS overproduction due to hyperglycaemia can be mediated by the increase of Ca^2+^ uptake into cardiomyocytes of diabetic rats. In turn, the altered Ca^2+^ levels have also been recognized as a cause determining cardiac dysfunction independently from coronary artery disease or macroangiopathy [29,30], suggesting that this imbalance might constitute a further key event leading to the impairment of the myocardium.

We also demonstrated that, in a diabetic heart, NADPH oxidase activation was accompanied by the overexpression of both TSPO and VDAC1, which are localized at the outer mitochondrial membrane. In response to a moderate oxidative stimulus, overexpressed TSPO can form a complex with VDAC1 [31], in order to prevent intra-mitochondrial toxic accumulation of calcium [8,32]. In contrast, under more severe conditions of oxidative stress, TSPO and VDAC1 complex with other proteins involved in the formation of the mitochondrial transition pore, promoting apoptosis [33]. The detection of unchanged levels of apoptotic pathway markers, in our experiments, can confirm a possible saturation of calcium buffering power mediated by mitochondria, which, on the other hand, can cause an enhancement of calcium levels in the cytosol [8].

Although further studies are needed to measure mitochondrial and cytosolic calcium, our hypothesis is strongly supported by the literature. Indeed, despite that TSPO overexpression is aimed to preserve mitochondrial calcium homeostasis, inhibiting its uptake through VDAC1 [34], on the other hand, it has been demonstrated that the down-regulation of mitochondrial calcium impairs calcium-dependent activity of specific respiratory chain proteins, finally affecting ATP production [35,36]. Additionally, TSPO over-activation as well as the TSPO/VDAC1 interaction have been proven to suppress mitochondrial ATP generation and to promote mitochondrial ROS synthesis [8,34]. In consequence of all these events, the most affected target of respiratory chain appears to be Complex I [37]. In accordance with these discoveries, in the NPD + STZ group, primarily, our results show the overexpression of mitochondrial NADPH oxidase, which can also inactivate mitochondrial respiratory chain Complex I. Furthermore, a reduced level of prohibitin, characterizing a diabetic heart, through the down-regulation of Complex I, might further contribute to the impairment of mitochondrial membrane potential [38].

It has been recognized that inactive-latent or pro-MMP-2 requires Ca^2+^ ion, at the active site, for maintaining its stability. Moreover, MMP-2 secretion in ECM requires its activation, which is mediated at cell surface level by a subfamily of membrane-associated MMPs (MT-MMP), which are up-regulated under cytosolic calcium enhancement [39]. Our results show a down-regulation of secreted MMP-2 in the NPD + STZ group as compared with the control, which was ablated after calcium chelation, suggesting that the hypothesized enhancement of cytosolic calcium triggered the down-regulation of active MMP-2, via MT-MMP overexpression.

The decreased activation of the secrete form of MMP-2 might appear controversial as the literature mostly reported its overexpression during DCM, indicative of ECM degradation, interstitial collagen deposition, and consequent promotion of the fibrosis development [40]. Our discordant result suggests that, initially, in response to described molecular mechanisms triggered by hyperglycaemia, the lack of beneficial ECM remodelling of the heart might confer a form of “rigidity” to cardiac tissue that contributes to the development of heart failure. To the best of our knowledge, this hypothesis might be supported only by little evidence highlighting decreased plasma concentrations of MMP-2 in diabetic patients [41]. Although several factors had been hypothesized by the authors to justify the variability of MMP-2 plasma levels in response to hyperglycaemia [42], we suppose that these differences might simply reflect the evolution of the remodelling process during DCM that, in the early stages, is characterized by MMP-2 down-regulation. In this view, in vivo studies support our hypothesis as it has been reported that, in the heart of diabetic animals, decreased MMP-2 mRNA levels as well as MMP-2 protein expression and activity were associated with enhanced levels of tissue inhibitors of metalloproteinase-2 (TIMP-2) [43]. According to our theory, this early MMP-2 downregulation is reverted during the progression of diabetic cardiomyopathy, causing an increased deposition of degraded collagen and the development of the fibrosis and apoptotic cell death. Indeed, at this late stage, the pharmacological inhibition of this process by ivabradine determines a normalization of MMP-2 activation, the prevention of apoptosis, and an improvement of cardiac dysfunction [44].

The diversified evolution of cardiac damage mediated by ROS further emerges through the demonstration that their concentration can also variously modulate the action of the different isoforms of MMP-2. Particularly, it has been proved that, in the setting of an acute redox injury, a considerable part of newly synthesized MMP-2 is activated, thus remaining within the cell associated with the sarcomeric proteins and determining the cleavage of several crucial components of the contractile apparatus [11,45]. On the other hand, under persistent oxidative stress and the subsequent activation of an alternative intronic promoter, the expression of the NT-MMP-2 isoform occurs, promoting the following: the induction of specific mitochondrial-nuclear stress signaling, the impairment of cardiomyocyte function, and finally ventricular hypertrophy. In this context, cardiomyocyte damage does not result from the immediate deterioration of contractile apparatus by intracellular MMP-2, but from dysfunctional mitochondria and myocardial inflammation that, lately, lead to apoptotic cell death [11].

Here, we have demonstrated that, in response to the slight production of O_2_^−^ by NADPH oxidase, NT-MMP-2 is able to translocate from the mitochondrion/endoplasmic reticulum (ER) contact site, named mitochondria-associated membranes (MAMs), within the mitochondria. As MAM is considered a key element in the control of ER/mitochondrial calcium flux [11], we hypothesize that the enhancement of cytosolic calcium, favoured by the formation of the TSPO/VDAC1 complex, represents the early event responsible for NT-MMP-2 translocation into mitochondria and the consequent ultrastructural damage of these organelles [46].

Thus, it is convincing that an adequate strategy, aimed to control ROS production, intracellular calcium homeostasis, and mitochondrial function, as well as to maintain physiological levels of secreted active MMP-2 in diabetic patients, will permit to prevent the progressive degeneration of myocardial tissue.

## 4. Materials and Methods

### 4.1. Animals and Study Design

Male Sprague-Dawley rats (160−180 g; Envigo Laboratories Italia, Italy) were housed and cared for in accordance with the guidelines of the University of “Magna Graecia”, Catanzaro, Italy, as well in accordance with the Italian regulations for the protection of animals used for experimental and other scientific purposes (D.L. 26/2014) and with European Economic Community regulations (2010/63/UE).

All rats were maintained under equal conditions of temperature (21 ± 1 °C), humidity (60 ± 5%), and light/dark cycle, and chow and water were available ad libitum. Rats fed a normocaloric diet (normal pellet diet (NPD), 2018 Teklad Global Diet, Envigo, Italy) ad libitum for one month were randomly divided in two experimental groups (*n* = 6 per group): (1) rats (*n* = 6) treated intraperitoneally (i.p.) with streptozocin (STZ, 35 mg/kg, Sigma Aldrich #S0130, Milan, Italy); (2) rats (*n* = 6) treated intraperitoneally (i.p.) with the vehicle (citrate buffer, pH 4.4, i.p).

Blood glucose levels were measured using a glucometer (MultiCare, Biochemical Systems International, Arezzo, Italy). The measurements were carried out just before and 7 days after the vehicle or STZ injection, and once a week for the following 8 weeks.

At the end of the study, the animals were sacrificed under Zoletil 80 mg/kg and Domitor anesthesia; organs and tissues were rapidly removed, weighed, and immediately frozen in liquid nitrogen for molecular analysis or placed in containers of embedding neutral medium for criostate (Killik 05-9801, Bio-Optica, Milan, Italy) for immunohistochemistry/immunofluorescence analyses.

### 4.2. Echocardiographic Analysis

Rats were anesthetized with 1.5% isoflurane in supine position, hair was removed from the chest, and left ventricle (LV) images were collected with a high resolution Micro-Ultrasound System (Vevo 2100, VisualSonics Inc. Toronto, Canada) equipped with a MS250 linear transducer (13–24 MHz).

Body temperature was maintained at 37 °C with a heating pad and cardiac frequency was monitored with electrocardiography ECG electrodes and maintained over 300 BPM during the entire echocardiographic analysis.

Serial B-Mode and M-mode echocardiographic images were taken in parasternal short and long axis view at the level of the midpapillary muscles to assess functional parameters, cardiac function, and dimensions. At sacrifice, hemodynamic parameters were collected.

### 4.3. Strain Analysis

Strain analysis was conducted using a speckle-tracking algorithm provided by VisualSonics (VevoStrain, VisualSonics, Toronto, Canada). Echocardiographic speckle-tracking based strain measures of myocardial deformation were obtained from 2D gray scale echocardiography images acquired from the parasternal short-axis view (PSLAX). Strain and strain rate were quantified in the radial axis.

### 4.4. Immunohistochemistry

Cryogenic medium-embedded fresh ventricular cardiac cryostat-cut sections, 5 μm in thickness, were used for immunolabeling and confocal microscopy. Left ventricle cross sections (*n* = 3 for each animal) were fixed in 4% paraformaldehyde for 30 min at room temperature (RT), washed with phosphate-buffered saline (PBS), and immunohistochemistry analysis was performed using the UltraVision Quanto Detection System HRP DAB (Thermo Fisher Scientific, Waltham, MA, USA). Briefly, cryosections were incubated with a hydrogen peroxide/unspecific protein site blocking buffer. After washing, the primary antibody (20 µg/mL, anti-TSPO, MBL BV-3044-3, Woburn, MA, USA; 1:100, anti-NADPH oxidase 4, Abcam ab109225, Cambridge, UK) was applied overnight at 4 °C. After a washing step, sections were further incubated with a secondary antibody formulation conjugated to an enzyme-labeled polymer, for 10 min at RT. The polymer complex is finally visualized with an appropriate substrate/chromogen following the manufacturer’s instructions using an optical microscope (Olympus BX53, Shinjuku, Tokyo, Japan). The intensity of TSPO and NADPH oxidase staining was assessed using Image J (NIH, Bethesda, MD, USA). Briefly, the images were acquired in identical light conditions and converted into RGB images, which were separated via colour deconvolution (red, blue, and green components), providing a means of separating TSPO or NADPH oxidase related signals from the overlapping regions. The green component identified 3,3′-Diaminobenzidine DAB staining, because, using the green filter, only “brown” areas of the slice were highlighted. Subsequently, the region of interest (ROI) was detected, applying a constant threshold value (65–138 intensity units) for all images, in order to always choose the same range of colours/brightness. By setting this threshold value, the software converted this selection of interest into a percentage.

### 4.5. Mitochondrial Superoxide Anion Detection

Ventricular cryosections were fixed in 4% paraformaldehyde for 30 min at RT. After a washing step, sections were incubated for 10 min with 5 µM of a red mitochondrial superoxide indicator (MitoSOX™, Thermo Fisher Scientific, Waltham, MA, USA). Red fluorescence (absorption/emission: 510/580 nm) related to superoxide anion formation was detected using a confocal laser scanning microscope (Leica TCS SP5, Leica Microsystems, Wetzlar, Germany).

### 4.6. Protein Extracts

In order to obtain total omogenate, frozen left ventricles were pulverized and 200 mg of sample was homogenized in 300 μL ice-cold lysis buffer (100 mM Tris pH 7.6; 300 mM KCl; 0.1% Triton X 100; protease inhibitor cocktail—P8340, Sigma, Milan, Italy and phosphatase inhibitor cocktail—524625, Calbiochem, La Jolla, CA, USA) and centrifuged at 20,000× *g* for 20 min at 4 °C. After the centrifugation, the supernatant was collected for the evaluation of Bcl-2 expression.

Cytosolic fraction was obtained through subcellular fractionation of 50 mg of frozen and pulverized left ventricles homogenized in 500 μL ice-cold lysis buffer (10 mM Tris–HCl pH 7.4; 320 mM sucrose; 10 μL/mL protease (Sigma P8340) and phosphatase inhibitor cocktails (Calbiochem 524625). Nuclei were sedimented by centrifugation (5 min, 1300× *g* at 4 °C) and the supernatant was centrifuged at 17,000× *g* for 10 min at 4 °C to pellet the mitochondria. The supernatant, corresponding to the cytosolic fraction, was collected, aliquoted, and stored at 80 °C for evaluating cytochrome C expression.

The mitochondrial pellets were re-suspended in 175 μL ice-cold lysis buffer with 1% Triton X-100, subjected to three freeze/thaw cycles, and then sonified for 30 s on ice. The mitochondrial suspensions were then centrifuged at 20,000× *g* for 30 min to remove insoluble material. The supernatants were collected, aliquoted, and stored at 80 °C. Mitochondrial fraction was used to evaluate MnSOD activity and was resolved to detect VDAC1 expression.

Protein concentrations of total homogenate and subcellular fractions were determined using the Bio-Rad DC protein assay (Quick start Bradford 1X Dye reagent, Biorad #500-0205, Hercules, CA, USA).

### 4.7. SOD Activity

Superoxide dismutase (SOD) activity was performed on mitochondrial fraction using a SOD determination kit (Sigma-Aldrich #19160, Milan, Italy) according to the manufacturer’s protocol. Briefly, sample solution was treated with water-soluble tetrazolium salt (WST), and then the mixture was heated for 20 min at 37 °C. The absorbance of formed formazan salt was recorded at 450 nm using a microplate reader. SOD activity was defined as the amount of the enzyme inhibiting WST reduction.

### 4.8. Western Blot Analysis

Proteins were heat denatured for 5 min at 95 °C in sample-loading buffer (500 mM Tris/HCl, pH 6.8; 30% glycerol; 10% sodium dodecyl sulfate; 5% β-mercaptoethanol; and 0024% bromophenol blu), and 20 μg of protein lysate was resolved by sodium dodecyl sulfate SDS–polyacrylamide gel electrophoresis and transferred to nitrocellulose membranes (Amersham protran 0.2 µm NC 10600001, Little Chalfont, UK). After incubation in blocking solution (Tris/HCl, pH 7.6 containing 0.1% Tween 20 and 5% non-fat dried milk), membranes were incubated with the following primary antibodies: anti-Bcl2 (1:500, Cell signaling #2876, Danvers, MA, USA), anti-Cytochrome C (1:2000, BD Biosciences 556433, Italy), anti-VDAC1 (1:1000, Abcam AB15895, Cambridge, UK), and anti-β-actin (1:1000, Sigma Aldrich A3853, Milan, Italy) overnight at 4 °C. Membranes were then washed in tris-buffered saline TBS (pH 7.6) with 0.1% Tween-20 and incubated with horseradish peroxidase-conjugated secondary antibodies (anti-rabbit antibody Pierce #31460 or anti-mouse antibody Pierce #31430, Invitrogen, Carlsbad, CA, USA) for 1 h at RT with shaking. Immunoblot scanning and analyses were performed using an imaging system (UVITEC Imaging Systems, Cambridge, UK). Quantification of the bands was performed using the ImageJ software (NIH, Bethesda, MD, USA).

### 4.9. Immunofluorescence

Ventricular cryosections were fixed in 4% paraformaldehyde for 30 min at RT; then, sections were washed with PBS and blocked for 30 min with GDB 1X (Gelatin Dilution Buffer 10X containing gelatin 2%, Triton X-100 0.6%, NaH_2_PO_4_ 40 mM, NaCl 0.9 M, pH 7.4). After the blocking step, sections were incubated overnight at 4 °C in a humidified chamber with anti-MMP-2 antibody (1:250; Abcam ab37150, Cambridge, UK). Then, sections were washed with PBS and incubated with a fluorescent secondary antibody (Alexa Fluor 488, Thermo Fisher Scientific, Waltham, MA, USA) for 1 h at RT.

Sections were washed and incubated for 10 min with 5 µM of MitoSOX™ (Thermo Fisher Scientific, Waltham, MA, USA) or 1 µM endoplasmic reticulum (ER)-Tracker™ (Blue-White DPX, Thermo Fisher Scientific, Waltham, MA, USA). Fluorescence was detected using a confocal laser scanning microscope (Leica TCS SP5, Leica Microsystems, Wetzlar, Germany).

### 4.10. MMP Precipitation and Gel Zymography

Frozen left ventricles were separately pulverized and homogenized in a 1:3 *w/v* ratio with a modified ice-cold TBS buffer (50 mM Tris-HCl; 150 mM NaCl; 5 mM CaCl_2;_ 0.05% Brij L23, pH 7.6; 0.02% NaN3; 1% Triton X-100; 10 μL/mL, freshly added protease and phosphatase inhibitor cocktails) and centrifuged at 14,000× *g* for 20 min at 4 °C.

Afterwards, 4 mg of total protein lysate for each sample was subjected to affinity precipitation with gelatin-conjugated Sepharose beads (Gelatine-Sepharose 4B, Amersham Biosciences, GE Healthcare, Milan, Italy), overnight at 4 °C. The bound proteins were eluted from the beads in TBS containing 10% dimethyl sulfoxide DMSO by shaking for 1 h at 4 °C. Ten microliters of extracted cardiac samples were diluted (1:1) in a non-reducing loading buffer (63 mM Tris, 10% glycerol, 2% SDS, 0.1% bromophenol blue, pH 6.8) and resolved on a 10% polyacrylamide gel copolymerized with 0.1% gelatin (gelatin from porcine skin Sigma Aldrich G8150, Milan, Italy). After electrophoresis, the gel was washed twice in 2.5% Triton X-100 solution for 30 min at room temperature, in order to remove SDS, and twice for 15 min with distilled water. Then, the gel was incubated for 40 h at 37 °C in a developing buffer containing 50 mM Tris–HCl, pH 7.78; 10 mM CaCl_2_; and 0.02% NaN_3_. After incubation, the gel was stained using 0.25% Brilliant Blue R (Sigma Aldrich B0149-5G, Milan, Italy) in 50% methanol and 10% acetic acid for 60 min and destained in 30% methanol and 10% acetic acid. Gelatinase activity was identified as clear zones against a blue background.

### 4.11. In Situ Zymography

In situ zymography with the MMP fluorogenic substrate dye-quenched-gelatin-fluorescein isothiocyanate DQ-gelatin-FITC (Molecular Probes, Eugene, OR) was performed on Optimal cutting temperature compound OCT-embedded fresh cardiac cryostat-cut sections. Ventricular sections were air-dried for 3 h at room temperature; then rehydrated in PBS; and incubated overnight at 37 °C with the quenched fluorogenic substrate DQ-gelatin-FITC (40 µg/mL) alone or in combination with 20 mM EDTA in Tris-buffered saline (TBS) containing 50 mM Tris-HCl, 150 mM NaCl, 5 mM CaCl_2_, 0.05% Brij L23, pH 7.6, 0.02% NaN3. After washing, sections were fixed with 2% paraformaldehyde in PBS for 5 min and nuclei were counterstained with 4′,6-diamidino-2-phenylindole DAPI (0.1 µg/mL, Sigma, Milan, Italy) for 15 min at room temperature. Mounted slides were examined by confocal microscopy (Leica TCS SP5) to detect the green fluorescence due to gelatinolytic activity. Scale bar: 50 µm.

### 4.12. Statistical Analysis

Data were analysed with GraphPad PRISM 6.0 (GraphPad Software, Inc., La Jolla, CA, USA). The results are shown as mean  ±  SEM. Normality was tested using the Kolmogorov–Smirnov test. Normally distributed data were analysed by *t*-test, whereas data without normal distribution were analysed using Mann–Whitney tests. A *p*-value < 0.05 was considered statistically significant.

## 5. Conclusions

Clinical management of diabetic cardiomyopathy represents an untargeted goal in therapy because of poor understanding related to the molecular mechanisms underlying the impairment of heart structure and function. To date, the aim of pharmacological research has been principally focused on the process of cardiac remodelling and, in particular, on the identification of those reactions leading to extracellular matrix degradation and fibrotic heart; on the other hand, the role of cardiomyocyte impairment still remains a little explored aspect in the pathogenesis of diabetic cardiomyopathy. Our results focus on the importance of dysfunctional cardiomyocyte, highlighting the central role of NT-MMP-2. In particular, we hypothesize that the direct cardiomyocyte injury as well as the inhibition of the early ECM remodelling by MMP-2 can determine a particular phenotype of the myocardial tissue, which becomes more “frail” and less reactive in counteracting the consequences of the hyperglycemic insult.

Overall, these data suggest that the possibility to detect and prevent the onset and progression of heart failure, through discovering novel biomarkers and therapeutic targets, will permit to ameliorate the management of the diabetic patients.

## Supplementary Materials

The following are available online at https://www.mdpi.com/1422-0067/21/20/7432/s1, Figure S1: Time course of blood glucose levels, Figure S2: MMP-2 gelatinolytic activity (upper panels) and MMP-2 gelatinolytic activity plus 20 mM EDTA (lower panels).
