# Supplementary material for: Role of TSPO/VDAC1 Upregulation and Matrix Metalloproteinase-2 Localization in the Dysfunctional Myocardium of Hyperglycaemic Rats
Source: Int J Mol Sci. 2020 Oct 9;21(20):7432. doi: 10.3390/ijms21207432 (PMC7587933; doi:10.3390/ijms21207432)
Supplement: Supplementary file 1 [file ijms-21-07432-s001.pdf]

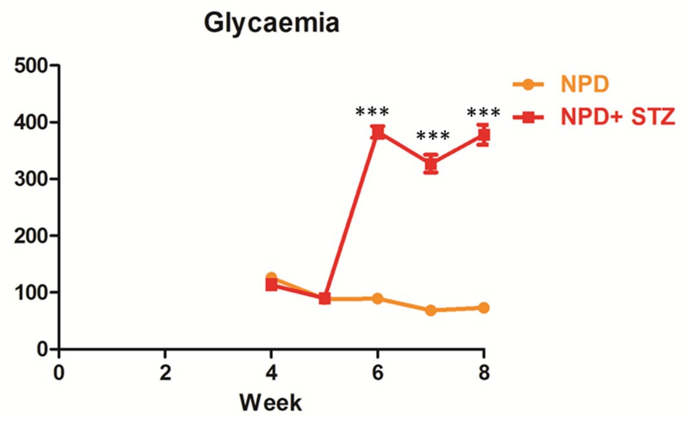

Supplementary Figure S1. Time course of blood glucose levels.

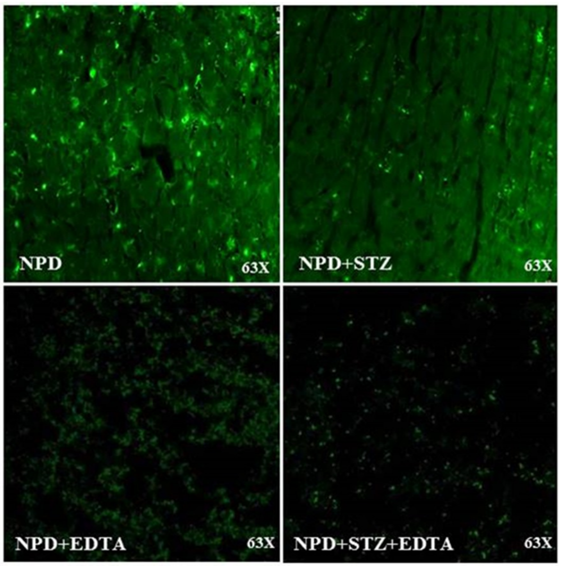

Supplementary Figure S2. MMP-2 gelatinolytic activity (upper panels) and MMP-2 gelatinolytic activity plus 20 mM EDTA (lower panels).
